# Depression and anxiety in patients with uveal melanoma undergoing curative proton treatment—A prospective study

**DOI:** 10.1002/cnr2.1780

**Published:** 2023-01-13

**Authors:** Christopher Rabsahl, Dirk Boehmer, Alexander Boeker, Ulrich Gauger, Ute Goerling, Johannes Gollrad

**Affiliations:** ^1^ Department of Radiation Oncology Charité – Universitätsmedizin Berlin Germany; ^2^ Department of Ophthalmology Charité – Universitätsmedizin Berlin Germany; ^3^ Statistical Consultant Berlin Germany; ^4^ Department of Psycho‐Oncology CCCC, Charité – Universitätsmedizin Berlin Germany

**Keywords:** anxiety, depression, prognosis, proton beam therapy, quality of life, uveal melanoma

## Abstract

**Objective:**

We prospectively addressed whether patient characteristics, oncological outcomes, or metastatic risk impacted depression and anxiety in patients undergoing curative proton treatment for uveal melanoma (UM).

**Methods:**

We assessed patient‐reported outcomes regarding anxiety (GAD‐7) before and 2 years after proton therapy and depression (PHQ‐9) before, 1, and 2 years after proton therapy. We performed descriptive statistics and used linear mixed effect modeling to analyze how the oncological outcome and baseline characteristics impacted anxiety and depression scores.

**Results:**

Of 130 (65 female) patients included, six developed metastatic disease and three died during the 2‐year follow‐up. The mean anxiety declined from 5.86 (SE = 0.56) at baseline to 3.74 (SE = 0.46) at 2 years (*β* = 2.11; SE = 0.6; *p* < .001). Depressive symptoms decreased moderately from 4.36 (SE = 0.37) at baseline to 3.67 (SE = 0.38) 2 years later. Patients with unfavorable metastatic risk or disease progression had elevated anxiety and depression scores. Although female patients reported overall higher anxiety scores, both sexes recovered substantially and to a similar extent during the 2‐year follow‐up (*β* = 2.35; SE 0.87; *p* = .007 vs. *β* = 1.88; SE = 0.60; *p* = .002). A trend for prolonged depressive symptoms was observed in patients living alone compared to patients living with family members 1 year after the treatment (*M* = 5.04 [SE = 0.85] vs. *M* = 3.73 [SE = 0.31], *β* = 1.32; SE = 0.92; *p* = .152). Patients with high baseline anxiety levels showed initially more severe depressive symptoms, which improved significantly during follow‐up (*β* = 1.65; SE = 0.68; *p* = .017).

**Conclusion:**

Most patients undergoing proton therapy for UM experienced mild, transient depressive symptoms and anxiety. Patients with high pre‐treatment anxiety, unfavorable prognoses, and patients living alone may be more vulnerable to prolonged depressive symptoms. To these patients a more tailored support could be offered at an early stage of the disease.

## 1 INTRODUCTION

Uveal melanoma (UM) is the most common primary cancer of the eye; its annual incidence is 5.1 cases per 1 million.^1^ At diagnosis, some patients with UM are asymptomatic, but others present with typical symptoms, including blurred vision, visual field defects, and photopsia.^2^ The long‐term survival of patients with UM is largely related to metastatic progression, which occurs in up to 50% of patients, despite excellent local control rates.^3^ The risk of metastasis can be precisely determined by the genetic profile of the tumor, particularly when chromosome 3 is missing (i.e., monosomy 3).^4^, ^5^, ^6^

Historically, enucleation was the standard of care for non‐metastatic UM. Over the last few decades, conservative radiotherapeutic procedures have emerged, such as proton beam therapy and brachytherapy, which are equally effective, eye‐conserving treatments.^7^, ^8^ Moreover, for large, or more centrally located UM, proton beam therapy has been established as a highly precise local treatment, and it has an excellent (over 97%) 5‐year tumor control rate.^9^ This non‐invasive procedure is typically administered on consecutive days with a total irradiation dose of 60 Cobalt Gray equivalents. In recent years, enucleation and radiotherapeutic procedures for treating UM have demonstrated similar effectiveness regarding oncological outcome. Consequently, in patients with UM, quality of life aspects, including disease‐related depression and anxiety, have become increasingly important.

Previous studies that examined depression and anxiety in UM have shown broad heterogeneity in study design, treatment modalities, assessment points, and prognostic genetic testing.^10^, ^11^, ^12^, ^13^, ^14^, ^15^, ^16^ In general, most studies reported that anxiety declined, and depression declined or remained stable during follow‐up, based on the Hospital anxiety and Depression Scale (HADS). However, very few studies have reported prospective data on anxiety and depression that included pre‐treatment assessments. In one study, Brandberg et al. found a decline in anxiety and stable depression levels in 99 patients at 1 year after enucleation or brachytherapy. In most of these studies, treatment with proton beam irradiation was frequently not addressed or it was administered to a small minority of the cohort, although several studies have demonstrated the relevance of treatment modality for the quality‐of‐life outcomes of patients with UM.^11^, ^12^, ^14^, ^15^, ^16^, ^17^ To our knowledge, Moschos et al.^14^ performed the only study that specifically investigated depression after proton beam irradiation in patients with non‐metastatic UM. In that cross‐sectional study, depression was assessed with the Patient Health Questionnaire‐9 (PHQ‐9) module, which measures major depressive disorders. Patients were assessed at 5 years after completing treatment and the scores were compared to scores from a healthy control cohort. Interestingly, those patients showed exceptionally severe depression, compared to the results reported in the existing literature on patients with non‐metastatic UM.

To our knowledge, no prospective studies have been published on depression and anxiety in patients with UM treated with proton beam radiation, which included intra‐subject reference data at baseline. The present prospective study aimed to determine whether proton beam treatment for UM affected depression and anxiety and to identify predictors of vulnerable patients that might benefit from early support.

## 2 METHODS

### 2.1 Patient recruitment and study design

Between May 2019 and January 2020, a total of 183 patients diagnosed with UM and eligible for proton irradiation were referred to our ophthalmologic department. Eligibility criteria for proton therapy were a central tumor location or a tumor height that exceed 5 mm. Patients were excluded from the study when they had a tumor confined to the iris, metastasis, a tumor recurrence, or insufficient German language skills. All patients meeting the inclusion criteria were consecutively invited to participate in our prospective quality of life study.

Patients were recruited 6–14 days before the start of proton therapy during inpatient preparations for the treatment. After written informed consent was given, patients received baseline questionnaires, which were completed and returned the same day. Follow‐up questionnaires were sent to patients 1 (PHQ‐9) and 2 years (PHQ‐9 and GAD7) after proton therapy, after a telephone reminder 1 week before the questionnaires were sent.

If no response was received within 2 weeks, we once again reminded the patients via phone call and, if applicable, recorded the reasons for a dropout as reported by the patient. The study was approved by the local Ethics Committee and conducted in accordance with the Declaration of Helsinki.

### 2.2 Measures and assessment points

The PHQ‐9 is the depression module of the Patient Health Questionnaire (PHQ). It was assessed at baseline and at 1 and 2 years after treatment. The questionnaire referred to the 2 weeks prior, and it included nine items that were scored from 0 (not at all) to 3 (nearly every day). Thus, scores ranged between 0 and 27 points. The PHQ‐9 was shown to be a reliable instrument for measuring the severity of depressive symptoms. Scores of 5, 10, 15, and 20 indicated mild, moderate, moderately severe, and severe clinical concern for depression, respectively. Scores ≥10 indicated a major concern for depression, with a sensitivity and specificity of 88%.^18^

The likelihood of the presence of generalized anxiety disorder was assessed with the General Anxiety Disorder‐7 (GAD‐7) questionnaire. It was assessed at baseline and at the 2‐year follow‐up. The questionnaire was validated for screening generalized anxiety disorder and estimating severity.^19^ It consisted of seven items that that were scored as 0 (not at all), 1 (on single days), 2 (on more than half of the days), or 3 (almost every day). All questions referred to subjective impairments due to complaints experienced in the last 2 weeks. The total score ranged from 0 to 21 points. A score ≥10 indicated a clinical concern for generalized anxiety disorder. Furthermore, scores of 5, 10, and 15 indicated mild, moderate, and severe concern for anxiety symptomatology, respectively.^19^

Sociodemographic data were collected with a self‐reported questionnaire completed before proton beam radiation. Tumor characteristics were obtained from a clinical database. Information on the clinical course and psychological support provided during follow‐up was supplemented with a self‐reported questionnaire completed 2 years after treatment.

### 2.3 Statistics

Statistical analyses were performed with R, version 4.1.0 (The R Foundation for Statistical Computing, Vienna, Austria) with the packages *lme4* (version 1.1.27.1) and *emmeans* (version 1.6.2.1). Before analyses, missing data were treated with multiple imputation by Chained Equations using the *mice* package (version 3.14.0). The missing values we predicted by implementing the 2‐level *2l.pan‐method* using five iterations and generating 30 datasets. Predictive variables included age, sex, living with family, having kids, GAD‐7, and PHQ‐9.

The impact of variables on changes in depression and anxiety over time were studied by implementing age‐ and sex‐adjusted linear mixed effects models with a random intercept relating to the patient ID (1| ID). The final models were fitted using the lmer function (lme4) with restricted maximum likelihood estimation (REML) and providing the default covariance structure. In all models, baseline anxiety was implemented as a categorized variable (low risk for generalized anxiety disorder: score <4, high risk for generalized anxiety disorder: score ≥4). Regarding the outcome variable depression, age, sex, time, living situation * time and baseline anxiety * time were added as fixed effects and patient ID was set as random effect. Regarding the outcome variable anxiety, age, sex * time, and living situation * time were included as fixed effects and patient ID was set as random effect. The complete model formulas and parameters are provided as Supplementary Material. The variable selection was based on previous clinical observations in UM patients showing a significant influence of anxiety on the individual perception of somatic complaints (GAD‐7). Further predictors were not included into the final models because they either represented a small sub‐cohort (subgroups *n* ≤ 6) or had a minimal and non‐significant effect size and did not contribute to the overall performance of the models (having children). Post‐hoc analyses included pairwise comparisons and contrasting of estimated marginal means between different time points and for different subgroups using the *emmeans* package (version 1.6.2.1). Changes were considered significant for *p*‐values ≤.05.

## 3 RESULTS

### 3.1 Study cohort

Overall, 160 patients were eligible for proton therapy and met the inclusion criteria. Of these, 28 patients could not be contacted before the treatment and one patient declined participation. Finally, 130 patients (65 females, mean age 59.1 years, SD = 13.68) completed at least one baseline questionnaire and were included in the analysis. Before proton beam therapy, questionnaire response rates were 99.2% (129 patients) for the PHQ‐9 and 100.0% (130 patients) for the GAD‐7. At 1 year after treatment, 99 patients (76.2%) completed the PQH‐9. At 2 years after the treatment, 91 patients (70.0%) completed the PHQ‐9 and GAD‐7 questionnaires. Patient and tumor characteristics are summarized in Table 1.

**TABLE 1:** Baseline characteristics of patients

Characteristics	All patients	Male	Female
Patients, *n*	130	65	65
Mean age, years (range)	59.1 (20–84)	59.8 (30–84)	58.4 (20–80)
Tumor sub‐site			
Uvea only (without ciliary body involvement)	116 (89.2%)	61 (93.8%)	55 (84.6%)
Ciliary body only	2 (1.5%)	1 (1.5%)	1 (1.5%)
Combined ciliary body and uvea	12 (9.2%)	3 (4.6%)	9 (13.8%)
Iris only	0 (0%)	0 (0%)	0 (0%)
Social situation			
Living alone	21	6	15
Living in a social setting	109	59	50
Children	99	48	51
No children	31	17	14

### 3.2 Missing questionnaire data

Twenty‐one patients with missing questionnaire data could not be contacted by phone and we have no information why they discontinued their participation. However, according to our clinical database, most of these patients (*n* = 14, 67%) were alive 2 years after proton therapy. Of the remaining 18 patients with missing data, 3 had died, 8 indicated that they either had not received questionnaires (*n* = 2), had already sent the questionnaires (n = 4), or had forgotten to complete them (*n* = 2). Three participants stated that they had no time to complete the questionnaires, one was no longer interested in participating, three patients no longer wished to participate due to illness other than UM, and two gave private reasons.

### 3.3 Anxiety

**FIGURE 1:**
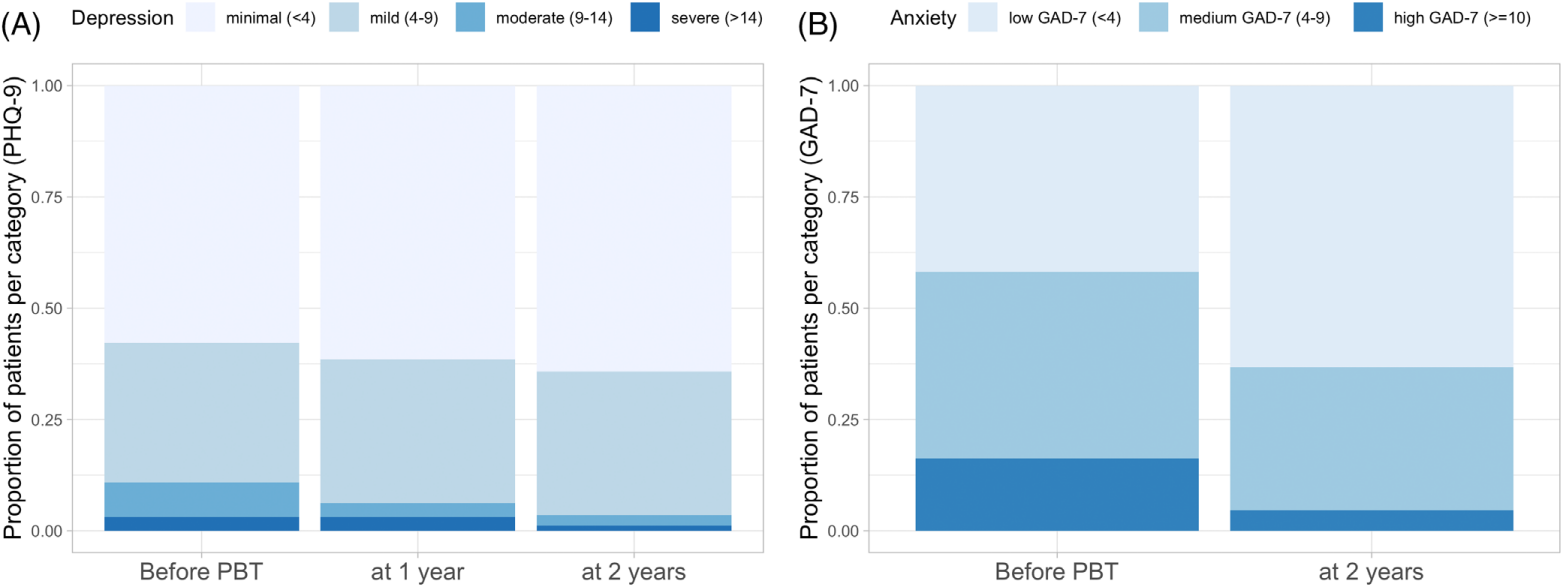
Proportion of patients in each category of (A) depression and (B) anxiety before proton beam therapy (PBT), and at 1 and 2 years after treatment

**FIGURE 2:**
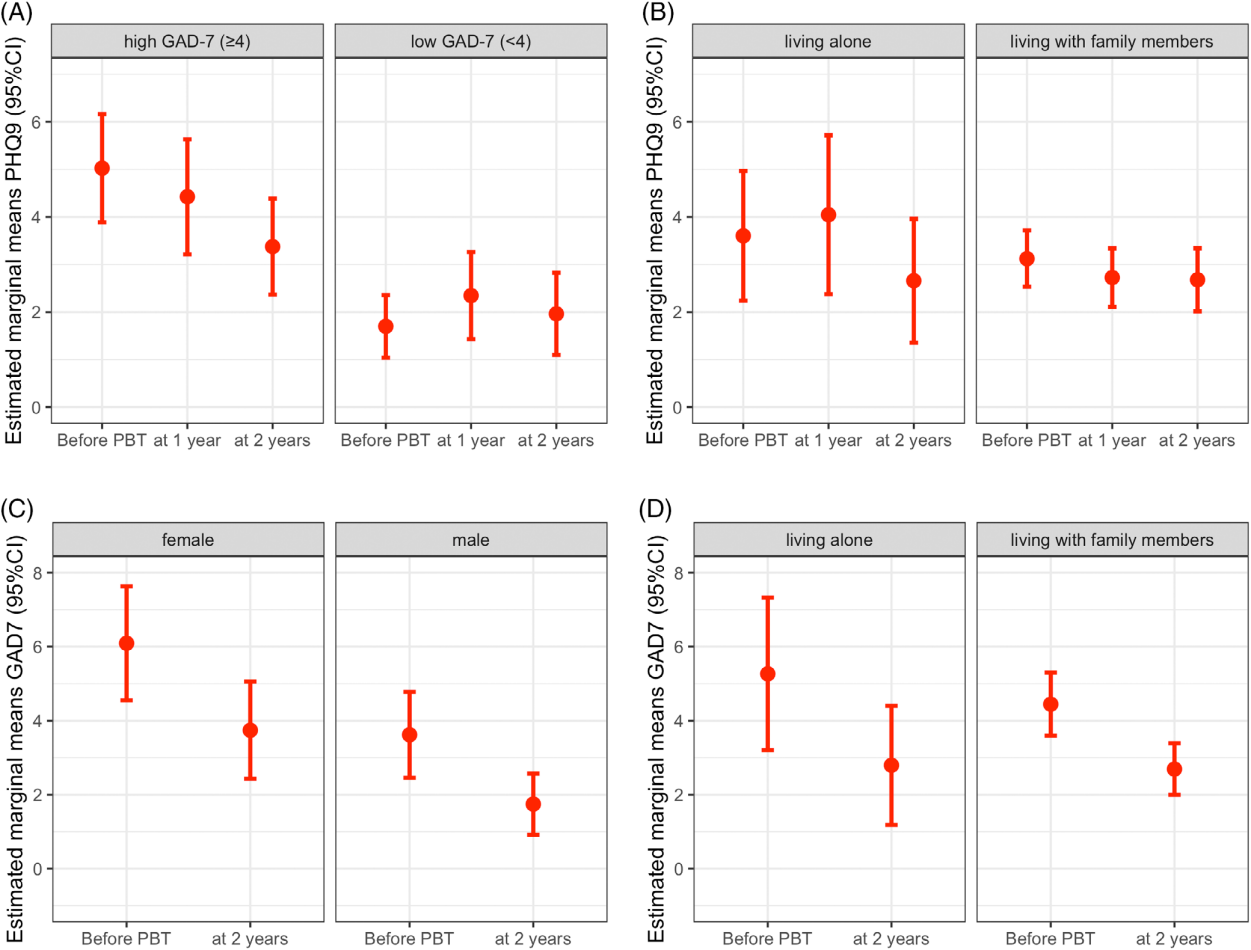
Estimated marginal means of depression (A, B) before, 1, and 2 years after proton beam therapy (PBT) comparing subgroups regarding (A) pre‐treatment anxiety and (B) living situation. Results are averaged over the levels of sex and living situation (A) and sex and baseline GAD‐7 (B), respectively. Below: Marginal means of anxiety (C, D) before and 2 years after PBT comparing subgroups regarding (C) sex and (D) living situation averaged over the levels of living situation (C) and sex (D)

Before proton beam therapy, the study cohort had a mean anxiety score of 5.86 (CI 4.76–6.95). Within 2 years, estimated marginal means of anxiety declined significantly to 3.74 (CI 2.85–4.64), when averaged over the levels of sex and living situation (*β* = 2.11; SE = 0.6; *p* < .001; Table 2). At baseline, 21 patients (16.3%) showed signs of moderate to severe anxiety, according to the GAD‐7 (≥10). At 2 years after therapy, only four patients (4.6%) reported these symptoms (Figure 1). Female patients showed higher anxiety scores compared to male patients at baseline (*M* = 7.09 [SE = 0.78] vs. *M* = 4.62 [SE = 0.59]; *β* = 2.47; [SE = 0.83]; *p* = .003) and 2 years later (*M* = 4.74 [SE = 0.67] vs. *M* = 2.74 [SE = 0.42]; *β* = 2.0; *p* = .002). However, anxiety recovered for both sexes to a similar extent when contrasting marginal means over time intervals for both sexes (*β* = 2.35 [SE = 0.87] vs. *β* = 1.88 [SE = 0.6]; *p* < .007; Figure 2). No effects on anxiety outcome were observed for age and living situation Supplementary Material.

**TABLE 2:** Anxiety and depression at different timepoints

Mean Score	Before PBT	At 1 year	At 2 years	Estimate	*p*
GAD‐7 score	5.86 [4.76–6.95]	–	3.74 [2.85–4.64]	2.11	<.001
PHQ‐9 score	4.36 [3.63–5.09]	4.38 [3.51–5.26]	3.67 [2.93–4.41]	0.693	.140

*Note*: Estimated marginal means with 95% confidence intervals for depression (PHQ‐9) and anxiety (GAD‐7) at different timepoints, averaged over the levels of sex, living situation and pre‐treatment anxiety (PHQ‐9 only). Changes over time intervals were tested by contrasting estimated means before and 2 years after proton treatment. No. of patients: *n* = 130. No. of observations *n* = 260 (GAD‐7) and *n* = 390 (PHQ‐9).

### 3.4 Depression

Before proton beam therapy, the study cohort had a mean depression score of 4.36 (CI 3.63–5.09) and showed a trend for improvement of depression to 3.67 (CI 2.93–4.41) at 2 years (*β* = 0.693; SE = 0.47; *p* = .140; Table 2). At baseline, 14 patients (10.9%) met the criterion for a high likelihood of a major depression (PHQ‐9 ≥10). During follow‐up, the proportion of these patients declined to 6.2% at 1 year and 4.5%, at 2 years after treatment (Figure 1).

By introducing baseline anxiety and time as an interaction term, we found the course of depression to be dependent on the pretreatment anxiety level: While depression remained at low levels for patients with low baseline anxiety (GAD‐7 < 4), we observed substantially higher depressive symptoms at baseline in patients with severe baseline anxiety, which recovered from 6.02 (CI 4.89–7.16) at baseline to 4.37 (CI 3.37–5.38) at 2 years (*β* = 1.65; SE = 0.68; *p* = .017; Figure 2).

At baseline, patients living alone showed similar means for depression scores than patients that lived with family or relatives (4.60 [CI 3.24–5.97] vs. 4.12 [CI 3.53–4.71]). Interestingly, patients who lived alone had a delayed recovery from their depressive symptoms, showing higher mean depression scores compared to patients living with family members 1 year after treatment (5.04 [CI 3.37–6.72] vs. 3.73 [CI 3.11–4.34]; *β* = 1.32; SE = 0.92; *p* = .152). After 2 years, depression scores normalized to a comparable level in both subgroups (3.66 [CI 2.36–4.96] vs. 3.68 [CI 3.02–4.34], Figure 2). No effects on depressive symptoms were observed for sex and age Supplementary Material.

### 3.5 Impact of kids

Patients without children showed a trend for increased depression (4.27 SD = 3.43 vs. 3.52 SD = 3.27) and anxiety (3.88 SD = 4.11 vs. 3.71 SD = 3.35) compared to patients with children, at 2 years after treatment. However, having children had no relevant role with respect to depression or anxiety in exploratory mixed effect models.

### 3.6 Impact of oncological outcome

Two years after proton therapy, one patient had a local recurrence (1.1%) and six patients developed distant metastases (6.6%). Overall, three patients died (3.3%): two due to distant metastases. Patients with metastases tended to have higher depression scores (5.33, SD = 3.51 vs. 3.66, SD = 3.49) and slightly greater anxiety scores (4.33 SD = 4.04 vs. 3.78 SD = 3.53) than those without metastases.

### 3.7 Impact of prognostic genetic testing

In our cohort, only 13 patients (14.3%) underwent optional prognostic cytogenetic testing to assess the patients’ future risk of metastatic disease. Of these, 6 (46.2%) patients were aware of a favorable prognosis (disomy 3), and 3 (23.1%) patients were aware of an unfavorable prognosis (monosomy 3). Four (30.8%) patients could not recall the test result. Patients with unfavorable prognoses showed slightly increased depression (4.67 SD = 3.79 vs. 4.33 SD = 3.93) and considerably increased anxiety (7.67 SD = 7.51 vs. 2.83 SD = 3.37), compared to patients with favorable testing results.

### 3.8 Patient‐reported psychological support during follow‐up

Although we encouraged all patients to receive psycho‐oncological care, only 12 patients (13.2%) reported receiving professional psychological support at some point during the 2‐year follow‐up. Of these, only one patient was currently in psycho‐oncological care at 2 years after treatment. Patients that underwent professional psychological support reported increased PHQ‐9 scores (7.44 SD = 5.73 vs. 3.18 SD = 2.82) and higher GAD‐7 scores (5.6 SD = 4.6 vs. 3.49 SD = 3.35) compared to patients that did not receive psychological care.

## 4 DISCUSSION

### 4.1 Main findings

This was the first prospective study to address the course of depression and anxiety in newly diagnosed patients with UM that were uniformly treated with proton beam therapy. Our results indicated that depression and anxiety represented a significant burden among patients with UM, prior to the irradiation procedure. During follow‐up, we observed a decline in depressive symptoms and a significant reduction in anxiety. Two years after the primary treatment, mean symptom scores were within the range of German normative values for depression (*M* = 2.91; SD = 3.52) and anxiety (*M* = 2.95; SD = 2.95), although direct comparisons are limited due to substantial differences regarding age and sociodemographic population structure.^20^, ^21^

### 4.2 Anxiety

Our results confirmed the findings from several previous studies that described elevated peri‐interventional anxiety and depression in patients undergoing primary treatment for UM.^10^, ^12^, ^13^, ^15^, ^16^ Moreover, the significant decline in anxiety observed in our patients was consistent with existing literature, which uniformly showed similar anxiety relief after various primary treatments.^10^, ^13^, ^15^, ^16^, ^22^ Interestingly, the reduction in anxiety appeared to occur shortly after treatment. For example, Suchocka‐Capuano et al.^16^ observed a significant decline in anxiety in a cohort of 69 patients only one month after therapy. Most of those patients were treated with proton beam therapy. In addition, in a preliminary study, we reported declines in *fear of recurrence* immediately and 3 months after proton beam therapy, compared to pre‐therapeutic assessments.^23^ Apparently, for most patients, anxiety promptly and significantly recedes after completing a primary treatment, and it continues to decline over time.^13^

However, previous studies reported persistently high anxiety over the long term, after primary treatment, in a particular sub‐cohort of patients.^13^, ^24^, ^25^ In our study, particularly in female patients and patients with unfavorable prognoses, anxiety remained higher than the level observed in a corresponding control group. The observation that anxiety levels were significantly elevated in female, but not male, patients was consistent with studies on normative values.^21^ Although elevated anxiety seems reasonable among patients with an unfavorable prognosis or metastatic progression, in a previous study, the monosomy 3 status was not identified as a predictor of anxiety at 24 months after therapy.^25^

### 4.3 Depression

Previous studies have been more inhomogeneous regarding the development of long‐term depression in patients with UM after primary treatment. For example, Brandberg et al.^10^ found persistently elevated depression levels 1 year after treatment, compared to pretreatment assessments, in 99 patients that underwent enucleation or brachytherapy, based on the HADS questionnaire. In contrast, Schuermeyer et al.^15^ observed stable depression levels in 96 patients, and 9% of patients displayed either possible or probable depression within a 1‐year follow‐up. In another study, Hope‐Stone et al. evaluated 411 patients with the HADS questionnaire at 6, 12, and 24 months after various primary treatments (17.5% were treated with proton beam irradiation) and compared the results to normative values. Interestingly, those authors observed less depression in patients with UM, compared to a normal population, at all assessment points. They concluded that cancer survivors may be more aware of enjoying and valuing their lives, compared to individuals without cancer.^12^ It was difficult to interpret these inconsistent findings and directly compare them to our results, due to the use of different assessment points, questionnaires, and treatment modalities.

In the study by Moschos et al.,^14^ 50 patients with non‐metastasized UM showed surprisingly high levels of mean depression scores (PHQ‐9: 10.18 ± 4.68) at 5 years after completing treatment. That finding contrasted with our findings and with previous reports that observed stable to declining depression after treatment.^10^, ^12^, ^15^ This discrepancy might partly be explained by the relatively high symptom severity of visual impairments in the cohort of Moschos et al.; indeed, severe symptomology has previously been associated with higher depression scores.^24^, ^25^ However, the lack of baseline data in the Moschos et al. study complicated the interpretation of the results.

In our study, female patients had elevated depression scores, compared to male patients, which is consistent with normative values.^20^ Patients living alone showed slightly higher depression than those living with relatives during the first year after treatment, but no differences were observed between patients with and those without children. These results suggested that an in‐house social support network might be important during the more vulnerable phases after diagnosis and treatment. However, these results must be interpreted with caution, as the sample size of patients living alone was relatively small (*n* = 21) and effects did not show a clear significance in our statistical models. Additional unknown confounding factors may have influenced our observation. Interestingly, "living alone" seemed to influence patient depression, but the level of anxiety differed only slightly between patients living alone and those living in more social settings.

Patients that received professional psychological support between the irradiation and the 2‐year assessment showed a trend for higher depression scores and increased anxiety. These findings suggested that some psychologically burdened patients—but not all—sought assistance. However, despite persistent problems, only one patient had continued to receive psychological treatment at 2 years after radiotherapy. In these patients, long‐term care may be recommended.

In general, our observed correlation between depression and anxiety confirmed the well‐known close relationship between these conditions.^15^ Moreover, our data suggested that, in addition to the correlation between depression and anxiety at baseline, baseline anxiety could predict the future course of depression at 24 months after treatment, even though, for most patients, anxiety had diminished at 24 months. Thus, the GAD‐7 questionnaire could be a useful tool for identifying vulnerable patients that might benefit from early support and assistance.

### 4.4 Limitations

The major strengths of this study were its prospective design and the homogeneous patient cohort, regarding primary treatment. Moreover, we assessed data before and after proton beam therapy, which made it possible to evaluate long‐term changes in depression and anxiety compared to pretreatment values. Finally, the overall questionnaire response rate was good, which provided sufficient data for reliable results.

One limitation of this study was the lack of survey data for anxiety at 1 year after irradiation. When the study was designed, we measured anxiety at baseline, and the 2‐year follow‐up was only introduced later in the course of the study. Another limitation was that few patients in our study cohort were affected by metastases or underwent prognostic genetic testing. Thus, some subgroups had small sample sizes, and we performed explorative analyses. Consequently, those findings should be interpreted with caution. Finally, the follow‐up period overlapped considerably with the peak of the COVID‐19 pandemic; thus, the pandemic may have biased the observed results on anxiety and depression.

## 5 CONCLUSION

During the first 2 years after initial diagnosis and curative proton beam therapy, both depression and anxiety improved steadily for most patients with UM. The long‐term burden of surviving patients was influenced by an unfavorable prognosis or metastatic progression over time, and it also appeared to be determined by the patient's individual vulnerability, expressed as pre‐treatment anxiety. Moreover, living with relatives appeared to be protective, particularly during the first year after treatment. Future studies with larger sample sizes may be needed to extend our understanding of the factors that influence the psychological well‐being of patients with UM.

## AUTHOR CONTRIBUTIONS

**Christopher Rabsahl:** Conceptualization (lead); data curation (lead); formal analysis (lead); investigation (lead); methodology (lead); project administration (supporting); resources (equal); software (equal); supervision (equal); validation (lead); visualization (equal); writing – original draft (lead); writing – review and editing (lead). **Dirk Boehmer:** Conceptualization (equal); data curation (equal); formal analysis (equal); investigation (equal); methodology (equal); project administration (equal); resources (equal); supervision (equal); writing – original draft (equal); writing – review and editing (equal). **Alexander Boeker:** Data curation (equal); investigation (equal); methodology (equal); validation (equal); writing – original draft (equal); writing – review and editing (equal). **Ulrich Gauger:** Formal analysis (lead); investigation (supporting); methodology (supporting); software (equal); visualization (supporting). **Ute Goerling:** Conceptualization (lead); formal analysis (equal); investigation (equal); methodology (equal); supervision (equal); writing – original draft (equal); writing – review and editing (equal). **Johannes Gollrad:** Conceptualization (lead); data curation (supporting); formal analysis (lead); investigation (lead); methodology (lead); project administration (lead); resources (equal); software (lead); supervision (lead); validation (lead); visualization (lead); writing – original draft (lead); writing – review and editing (lead).

## CONFLICT OF INTEREST

The authors have no relevant financial or non‐financial interests to disclose.

## ETHICS STATEMENT

This study was performed in line with the principles of the Declaration of Helsinki. Approval was granted by the Charité Ethics committee, Reference number: EA4/031/19. Informed consent was obtained from all individual participants included in the study.

## Supporting information


**Supplementary Material**: Click here for additional data file.

## Data Availability

The datasets used and analyzed in this study are available from the corresponding author on reasonable request.

## References

[1] Singh AD , Topham A . Incidence of uveal melanoma in the United States: 1973‐1997. Ophthalmology. 2003;110(5):956‐961. doi:10.1016/S0161-6420(03)00078-2 12750097

[2] Eskelin S , Kivela T . Mode of presentation and time to treatment of uveal melanoma in Finland. Br J Ophthalmol. 2002;86(3):333‐338. doi:10.1136/bjo.86.3.333 11864894 PMC1771030

[3] Kujala E , Makitie T , Kivela T . Very long‐term prognosis of patients with malignant uveal melanoma. Invest Ophthalmol Vis Sci. 2003;44(11):4651‐4659. doi:10.1167/iovs.03-0538 14578381

[4] Bronkhorst IH , Maat W , Jordanova ES , et al. Effect of heterogeneous distribution of monosomy 3 on prognosis in uveal melanoma. Arch Pathol Lab Med. 2011;135(8):1042‐1047. doi:10.5858/2010-0477-OAR1 21809997

[5] Damato B , Duke C , Coupland SE , et al. Cytogenetics of uveal melanoma: a 7‐year clinical experience. Ophthalmology. 2007;114(10):1925‐1931. doi:10.1016/j.ophtha.2007.06.012 17719643

[6] Prescher G , Bornfeld N , Hirche H , Horsthemke B , Jockel KH , Becher R . Prognostic implications of monosomy 3 in uveal melanoma. Lancet. 1996;347(9010):1222‐1225. doi:10.1016/s0140-6736(96)90736-9 8622452

[7] Diener‐West M , Earle JD , Fine SL , et al. The COMS randomized trial of iodine 125 brachytherapy for choroidal melanoma, III: initial mortality findings. COMS Report No. 18. Arch Ophthalmol. 2001;119(7):969‐982. doi:10.1001/archopht.119.7.969 11448319

[8] Rao PK , Barker C , Coit DG , et al. NCCN Guidelines Insights: Uveal Melanoma, Version 1.2019. J Natl Compr Cancer Netw. 2020;18(2):120‐131. doi:10.6004/jnccn.2020.0007 32023525

[9] Egger E , Schalenbourg A , Zografos L , et al. Maximizing local tumor control and survival after proton beam radiotherapy of uveal melanoma. Int J Radiat Oncol Biol Phys. 2001;51(1):138‐147. doi:10.1016/s0360-3016(01)01560-7 11516863

[10] Brandberg Y , Kock E , Oskar K , af Trampe E , Seregard S . Psychological reactions and quality of life in patients with posterior uveal melanoma treated with ruthenium plaque therapy or enucleation: a one year follow‐up study. Eye (Lond). 2000;14(Pt 6):839‐846. doi:10.1038/eye.2000.233 11584839

[11] Chabert S , Velikay‐Parel M , Zehetmayer M . Influence of uveal melanoma therapy on patients' quality of life: a psychological study. Acta Ophthalmol Scand. 2004;82(1):25‐31. doi:10.1046/j.1600-0420.2003.0210.x 14982042

[12] Hope‐Stone L , Brown SL , Heimann H , Damato B , Salmon P . Two‐year patient‐reported outcomes following treatment of uveal melanoma. Eye (Lond). 2016;30(12):1598‐1605. doi:10.1038/eye.2016.188 27589051 PMC5177756

[13] Melia M , Moy CS , Reynolds SM , et al. Quality of life after iodine 125 brachytherapy vs enucleation for choroidal melanoma: 5‐year results from the Collaborative Ocular Melanoma Study: COMS QOLS Report No. 3. Arch Ophthalmol. 2006;124(2):226‐238. doi:10.1001/archopht.124.2.226 16476893

[14] Moschos MM , Moustafa GA , Lavaris A , et al. Depression in choroidal melanoma patients treated with proton beam radiotherapy. Anticancer Res. 2018;38(5):3055‐3061. doi:10.21873/anticanres.12562 29715140

[15] Schuermeyer I , Maican A , Sharp R , Bena J , Triozzi PL , Singh AD . Depression, anxiety, and regret before and after testing to estimate uveal melanoma prognosis. JAMA Ophthalmol. 2016;134(1):51‐56. doi:10.1001/jamaophthalmol.2015.4343 26539659

[16] Suchocka‐Capuano A , Bredart A , Dolbeault S , et al. Quality of life and psychological state in patients with choroidal melanoma: longitudinal study. Bull Cancer. 2011;98(2):97‐107. doi:10.1684/bdc.2011.1300 21382791

[17] Hope‐Stone L , Brown SL , Heimann H , Damato B . Comparison between patient‐reported outcomes after enucleation and proton beam radiotherapy for uveal melanomas: a 2‐year cohort study. Eye (Lond). 2019;33(9):1478‐1484. doi:10.1038/s41433-019-0440-0 30988421 PMC7002659

[18] Kroenke K , Spitzer RL , Williams JB . The PHQ‐9: validity of a brief depression severity measure. J Gen Intern Med. 2001;16(9):606‐613. doi:10.1046/j.1525-1497.2001.016009606.x 11556941 PMC1495268

[19] Spitzer RL , Kroenke K , Williams JB , Lowe B . A brief measure for assessing generalized anxiety disorder: the GAD‐7. Arch Intern Med. 2006;166(10):1092‐1097. doi:10.1001/archinte.166.10.1092 16717171

[20] Kocalevent RD , Hinz A , Brahler E . Standardization of the depression screener patient health questionnaire (PHQ‐9) in the general population. Gen Hosp Psychiatry. 2013;35(5):551‐555. doi:10.1016/j.genhosppsych.2013.04.006 23664569

[21] Lowe B , Decker O , Muller S , et al. Validation and standardization of the generalized anxiety disorder screener (GAD‐7) in the general population. Med Care. 2008;46(3):266‐274. doi:10.1097/MLR.0b013e318160d093 18388841

[22] van Beek JGM , Buitendijk GHS , Timman R , et al. Quality of life: fractionated stereotactic radiotherapy versus enucleation treatment in uveal melanoma patients. Acta Ophthalmol. 2018;96(8):841‐848. doi:10.1111/aos.13823 30284368

[23] Gollrad J , Rabsahl C , Riechardt AI , et al. Quality of life and treatment‐related burden during ocular proton therapy: a prospective trial of 131 patients with uveal melanoma. Radiat Oncol. 2021;16(1):174. doi:10.1186/s13014-021-01902-6 34496895 PMC8425039

[24] Brown SL , Fisher PL , Hope‐Stone L , et al. Predictors of long‐term anxiety and depression in uveal melanoma survivors: A cross‐lagged five‐year analysis. Psycho‐Oncology. 2020;29(11):1864‐1873. doi:10.1002/pon.5514 32779313

[25] Brown SL , Hope‐Stone L , Heimann H , Damato B , Salmon P . Predictors of anxiety and depression 2 years following treatment in uveal melanoma survivors. Psycho‐Oncology. 2018;27(7):1727‐1734. doi:10.1002/pon.4715 29601654

