# Supplementary material for: Depression and anxiety in patients with uveal melanoma undergoing curative proton treatment—A prospective study
Source: Cancer Rep (Hoboken). 2023 Jan 13;6(4):e1780. doi: 10.1002/cnr2.1780 (PMC10075284; doi:10.1002/cnr2.1780)
Supplement: Supplementary file 1 — Supplementary Material: [file CNR2-6-e1780-s001.docx]

| Supplementary Material A: Final pooled linear mixed effect models for depression (PHQ-9) and anxiety (GAD-7) | | | | | | |
| --- | --- | --- | --- | --- | --- | --- |
|  | Depression (PHQ-9) | | | Anxiety (GAD-7) | | |
| **Predictors** | **Estimates** | **CI** | **p** | **Estimates** | **CI** | **p** |
| Age | 0.00 | -0.01 – 0.00 | 0.5 | 0.00 | -0.01 – 0.01 | 0.730 |
| Sex (male) | -0.20 | -0.40 – 0.00 | 0.056 | -0.43 | -0.71 – -0.15 | 0.003 |
| Sex (male)*Time (2 years) | - | - | - | -0.12 | -0.47 – -0.24 | 0.511 |
| Time (at 1 year) | -0.34 | -0.89 – 0.21 | 0.2 | - | - | - |
| Time (at 2 years) | -0.79 | -1.4 – -0.19 | 0.073 | -0.49 | -0.94 – -0.04 | 0.054 |
| Living with family members | -0.11 | -0.45– 0.22 | 0.5 | -0.14 | -0.51 – 0.23 | 0.512 |
| Living with family members  *Time (1 years) | -0.19 | -0.63 – 0.24 | 0.38 | - | - | - |
| Living with family members  *Time (2 years) | 0.12 | -0.34 – 0.57 | 0.62 | 0.11 | -0.36 – 0.59 | 0.640 |
| GAD-7 (Score <4) | -0.80 | -1.1 – -0.54 | <0.001 |  | | |
| GAD-7 (Score ≥4)*Time (1 year) | 0.32 | 0.01 – 0.65 | 0.057 |  |  |  |
| GAD-7 (Score ≥4)*Time (2 years) | 0.41 | 0.05 – 0.77 | 0.026 |  |  |  |
| **Model parameters** |  | | |  | | |
| N ID | 130 | | | 130 | | |
| Observations | 390 | | | 260 | | |
| m | 30 | | | 30 | | |
| Maximum likelihood est. | REML | | | REML | | |
| **Model formulas:** | lmer(phq9x ~ age +sex + fam_cat*time + gad7_1_cat2*time + (1\|ID), data = data) | | | lmer(gad7x ~ age + sex*time + fam_cat*time + (1\|ID), data = data) | | |
| **Variables:** phq9x: PHQ-9-score (outcome variable); gad7x: GAD-7-score (outcome variable); time: timepoint of assessment (3(2)-level, before treatment, (at 1 year,) at 2 years); fam_cat: living situation (2-level, living alone vs. living with family members); gad7_1_cat2: categorized variable for GAD-7 at baseline (2-level, low risk for generalized anxiety disorder: score <4, high risk for generalized anxiety disorder: score ≥4); ID: Subject ID | | | | | | |
